# Sex non-specific growth charts and potential clinical implications in the care of transgender youth

**DOI:** 10.3389/fendo.2023.1227886

**Published:** 2023-08-11

**Authors:** Eric Morris Bomberg, Bradley Scott Miller, Oppong Yaw Addo, Alan David Rogol, Mutaz M. Jaber, Kyriakie Sarafoglou

**Affiliations:** ^1^ Division of Endocrinology, Department of Pediatrics, University of Minnesota Medical School, Minneapolis, MN, United States; ^2^ Center for Pediatric Obesity Medicine, Department of Pediatrics, University of Minnesota Medical School, Minneapolis, MN, United States; ^3^ Department of Global Health, Rollins School of Emory University, Atlanta, GA, United States; ^4^ Division of Diabetes and Endocrinology, Department of Pediatrics, University of Virginia, Charlottesville, VA, United States; ^5^ Department of Experimental and Clinical Pharmacology, University of Minnesota College of Pharmacy, Minneapolis, MN, United States

**Keywords:** growth charts, growth, transgender, body mass index, pediatric obesity, nutrition surveys

## Abstract

**Introduction:**

The Centers for Disease Control and Prevention (CDC) and World Health Organization (WHO) created separate growth charts for girls and boys because growth patterns and rates differ between sexes. However, scenarios exist in which this dichotomizing “girls versus boys” approach may not be ideal, including the care of non-binary youth or transgender youth undergoing transitions consistent with their gender identity. There is therefore a need for growth charts that age smooth differences in pubertal timing between sexes to determine how youth are growing as “children” versus “girls or boys” (e.g., age- and sex-neutral, compared to age- and sex-specific, growth charts).

**Methods:**

Employing similar statistical techniques and datasets used to create the CDC 2000 growth charts, we developed age-adjusted, sex non-specific growth charts for height, weight, and body mass index (BMI), and z-score calculators for these parameters. Specifically, these were created using anthropometric data from five US cross-sectional studies including National Health Examination Surveys II-III and National Health and Nutrition Examination Surveys I-III. To illustrate contemporary clinical practice, we overlaid our charts on CDC 2000 girls and boys growth charts.

**Results:**

39,119 youth 2-20 years old (49.5% female; 66.7% non-Hispanic White; 21.7% non-Hispanic Black) were included in the development of our growth charts, reference ranges, and z-score calculators. Respective curves were largely superimposable through around 10 years of age after which, coinciding with pubertal onset timing, differences became more apparent.

**Discussion:**

We conclude that age-adjusted, sex non-specific growth charts may be used in clinical situations such as transgender youth in which standard “girls versus boys” growth charts are not ideal. Until longitudinal auxological data are available in these populations, our growth charts may help to assess a transgender youth’s growth trajectory and weight classification, and expectations surrounding these.

## Introduction

Because growth patterns and rates differ between sexes, the Centers for Disease Control and Prevention (CDC) and World Health Organization (WHO) created separate growth charts for girls and boys (1–3). The CDC 2000 growth charts for US children 2–19 years old, WHO 2006 growth charts for US children 0–2 years old and, in many countries the 1990 United Kingdom reference charts for children 0–5 years old, have been considered “reference” or “standard” for at least 15 years (3–5). However, scenarios exist in which this binary “girl versus boy” approach may not be ideal, including in care of non-binary youth or transgender youth undergoing transition consistent with their gender identity.

An increasing number of transgender youth are seeking medical care to establish treatment regimens geared towards developing physical characteristics congruent with experienced gender (6).

These interventions include gonadotropin releasing hormone agonists (GnRHa) to suppress puberty and reduce endogenous sex hormone production of one’s natal sex, and hormonal therapies (i.e., testosterone and estradiol) to induce secondary sex characteristics consistent with affirmed gender identity (7). Such therapies can impact anthropometrics, including linear growth, body mass index (BMI), and body composition (8, 9). For example, testosterone may increase muscle mass and BMI, while estradiol can lower waist-to-hip ratios due to hip fat deposition (10–12). Overall, the understanding of how hormonal interventions impact changes in pubertal growth spurt patterns is still in its infancy (13–15).

To monitor growth in non-binary youth or transgender youth and their response to medical interventions, currently clinicians have limited tools. It has been proposed that use of growth charts corresponding to both affirmed gender and genetic sex should/might be considered (10). For example, when evaluating growth parameters in a transgender male or female, the clinician must plot their height, height velocity, weight, and BMI on both the girls and boys CDC 2000 charts. Since all these growth parameters are sex dependent, assessments as to whether the growth response is appropriate become arbitrary as the determination is made by looking at the plot points of these parameters on both the male and female charts side-by-side. Because of the current challenges in monitoring growth in transgender youths, and the extended time it will take to develop gender-specific charts based on longitudinally collected data, an intermediate tool is needed.

Our group previously developed pubertal- and chronological-age adjusted growth charts and reference ranges using cross-sectional data from nationally representative US health surveys (e.g., National Health Examination Surveys (NHES), National Health and Nutrition Examination Surveys (NHANES) and Hispanic Health and Nutrition Examination Surveys (HHANES)) to assess how puberty affects anthropometrics including height, weight, and BMI (16–18). For example, we showed that considering race/ethnicity differences in pubertal timing affects shortness, tallness, and overweight/obesity prevalence quantification (16–18). Towards this end and utilizing similar techniques including use of pooled data from large US cross-sectional nationally-representative samples, we have now developed age-adjusted, sex non-specific growth charts and reference ranges for height, weight, and BMI, and z-score calculators for these parameters. In addition to sharing our growth charts and reference ranges, we exemplify how they can be used in clinical practice. Our goal in creating these tools is two-fold: to age-smooth growth across sexes in order to help clinicians monitor how one is growing as a “child” (sex non-specific) as opposed to specifically as a “girl or boy,” and as a research tool to monitor growth parameters until long-term auxological data can be systematically collected.

## Materials and methods

### Study population and data sources

To develop our age-adjusted, sex non-specific growth charts, reference ranges, and z-score calculators, we pooled data from five complex US cross-sectional nationally representative surveys that included children and adolescents: NHES II (1963-1965), NHES III (1966-1970), NHANES I (1971-1974), NHANES II (1976-1980), and NHANES III (1988-1994) (19–24). CDC/National Center for Health Statistics institutional review board approval and documented consent was obtained from participants.

We chose to use data from these surveys for two main reasons. First, all included nationally represented US youth. Second, CDC 2000 growth charts, the most widely used US reference for 2-20 year olds, are based upon data from these surveys, making our growth charts analogous (25). If we used more contemporaneous NHANES surveys, we could not compare our results to those from CDC 2000. We note that these surveys took place largely prior to the US obesity epidemic and, therefore, CDC 2000 growth charts and this analysis both excluded weights from NHANES III for youth ≥6 years to avoid upward shifts in weight- and BMI-for-age curves due to rising obesity prevalence.

### Study measures and inclusion criteria

Standing height to the nearest 0.1 cm and weight were measured by trained technicians following standardized protocols and using calibrated stadiometers and scales, respectively (26). In our analyses leading to creation of our growth charts, reference ranges, and z-score calculators, we included participants 2–20 years old to mirror CDC 2000 growth charts. We excluded children with missing data from any of the following: age, weight, height, and/or sex.

### Statistical analyses

We used the *Lambda, Mu, Sigma* (LMS) semi-parametric approach in a Generalized Additive Models for Location, Scale, and Shape (GAMLSS) technique to model growth (27). This approach has been used in many growth reference analyses, including those from the CDC and WHO (5, 28–30). Box-Cox Power distribution families in GAMLSS with additive age splines were used to calculate estimates of our sex non-specific height, weight, and BMI reference data tables (31, 32). We accounted for sampling weights to generate nationally representative chronological age-based growth charts for height, weight, and BMI without stratification by sex. Detailed statistical and visual diagnostic tools were used to select the best fitting model for generating reference data (33). Statistical analyses were conducted in R 3.6.0 (The R Foundation for Statistical Computing and Graphics, Vienna, Austria) and data management was performed using SAS 9.4 (SAS Institute, Cary NC, USA).

## Results

Data from 39,119 participants (49.5% female; 66.7% non-Hispanic White (NHW); 21.7% non-Hispanic Black (NHB)) were included in the development of our growth charts, reference ranges, and z-score calculators (see **Table 1**). Analytic sample sizes used to estimate our growth charts were large and robust.

**Table 1:** US Cross-Sectional Surveys Used to Develop Sex Non-Specific Growth Charts.

Survey	Years	Analytic Sample Size^*^ (N=39,119; Age Range= 2-20 Years Old)					
		Number of Participants	Age Range Years)	% Female	Race-Ethnicity		
					Group	N	%^†^
NHES II	1963-1965	7119	6–11 years	49.0%	NHW	6100	85.7%
					NHB	987	13.9%
					Other	32	0.4%
NHES III	1966-1970	6768	12– 17 years	47.6%	NHW	5735	84.7%
					NHB	999	14.8%
					Other	34	0.5%
NHANES I	1971-1974	7160	2-20 years	50.8%	NHW	5362	74.9%
					MA	71	1.0%
					NHB	1727	24.1%
					Other/multiracial	0	–
NHANES II	1976-1980	7355	2-20 years	48.5%	NHW	5998	81.6%
					MA	3742	2. 5%
					NHB	1177	16.0%
					Other/multiracial	0	-
NHANES III	1988-1994	10,717	2-20 years	51.0% (based on height)	NHW	2878	26.9%
					MA	3742	33.5%
					NHB	3587	34.9%
					Other/multiracial	510	4.8%
		4319	2-6 years^‡^	51.0% (based on weight)	NHW	1224	28.3%
					MA	1519	35.2%
					NHB	1360	31.5%
					Other/multiracial	216	5.0%

^*^Inclusion criteria: ages 2-20 years; Exclusion criteria: had missing data for age, height, weight, and/or sex.

^†^Percentages are unweighted.

**^‡^** Because of the rising obesity epidemic, the CDC 2000 growth charts, as well as ours, excluded weights from NHANES III youth >71 months old in order to avoid an upward shift in weight- and BMI-for-age curves due to rising overweight prevalence which would under-classifying overweight/obesity status.

NHES, National Health Examination Survey; NHANES, National Health and Nutrition Examination Survey; NWH, Non-Hispanic White; NHB, Non-Hispanic Black; MA, Mexican American.

**Figure 1:**
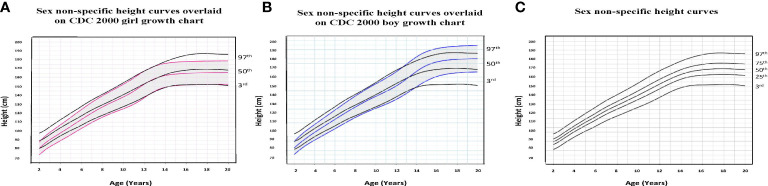
Comparison of age-adjusted sex non-specific height curves with CDC 2000 girl and boy age- and sex-adjusted height curves. **(A)** shows our age-adjusted sex non-specific height curves overlaid on the CDC 2000 girl age- and sex-adjusted height curves, highlighting the 3^rd^, 50^th^, and 97^th^ percentiles. **(B)** shows our age-adjusted sex non-specific height curves overlaid on the CDC 2000 boy age- and sex-adjusted height curve, highlighting the 3^rd^, 50^th^, and 97^th^ percentiles. **(C)** shows our age-adjusted sex non-specific height growth curves. Here, we highlight the 3^rd^, 25^th^, 50^th^, 75^th^, and 97^th^ percentiles given their clinical utility.

**Figure 2:**
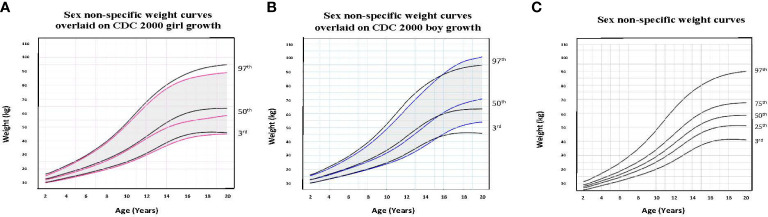
Comparison of age-adjusted sex non-specific weight curves with CDC 2000 girl and boy age- and sex-adjusted weight curves. **(A)** shows our age-adjusted sex non-specific weight curves overlaid on the CDC 2000 girl age- and sex-adjusted weight curves, highlighting the 3^rd^, 50^th^, and 97^th^ percentiles. **(B)** shows our age-adjusted sex non-specific weight curves overlaid on the CDC 2000 boy age- and sex-adjusted weight curve, highlighting the 3^rd^, 50^th^, and 97^th^ percentiles. **(C)** shows our age-adjusted sex non-specific weight growth curves. Here, we highlight the 3^rd^, 25^th^, 50^th^, 75^th^, and 97^th^ percentiles given their clinical utility.

**Figure 3:**
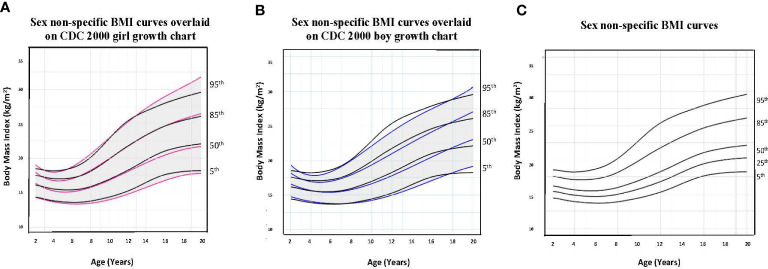
Comparison of age-adjusted sex non-specific body mass index (BMI) curves with CDC 2000 girl and boy age- and sex-adjusted BMI curves. **(A)** shows our age-adjusted sex non-specific BMI curves overlaid on the CDC 2000 girl age- and sex-adjusted BMI curves, highlighting the 5^th^, 50^th^, 85^th^, and 95^th^ percentiles. **(B)** shows our age-adjusted sex non-specific BMI curves overlaid on the CDC 2000 boy age- and sex-adjusted weight curve, highlighting the 3^rd^, 50^th^, 85^th^, and 95^th^ percentiles. **(C)** shows our age-adjusted sex non-specific BMI growth curves. Here, we highlight the 5^rd^ (underweight), 25^th^, 50^th^, 85^th^ (overweight), and 95^th^ (obesity) percentiles given their clinical utility.

To illustrate the clinical utility of our age-adjusted sex non-specific height, weight, and BMI growth charts, we overlaid them on the CDC 2000 girls and boys growth charts. Of note, when modeled using GAMLSS methods, the combined data (girls and boys) have a broader distribution resulting in a new set of percentiles that differ from those for either sex-specific cohort. Because of this, at certain age points, especially before puberty, the height and weight of children plotted on the sex non-specific growth chart may appear slightly taller and/or heavier than on the CDC 2000 boy and girl charts.* *As shown in **Figures 1**–**3**, the respective curves are nearly superimposable up to around 10 years of age (e.g., 10-12 years old) in terms of height and around 8 years of age (e.g., 8-10 years old) in terms of weight and BMI, after which differences become more apparent. For example, in a 5 year old, the median (p50) height on our age-adjusted sex non-specific curve is 111 cm, compared to 108 cm on the girl and 109 cm on the boy CDC 2000 charts. However, in a 15 year old the p50 height on our curve is 167 cm, compared to 162 cm and 170 cm on the CDC girl and boy charts, respectively.

As for median near adult height, our age-adjusted sex non-specific height curve shows p50 of 169 cm (range p3: 151 cm, p97: 186 cm), compared to 163 cm (p3: 151 cm, p97: 176 cm) and 177 cm (p3: 163 cm, p97: 190 cm) on the girl and boy CDC 2000 curves, respectively. A comparison of clinically useful percentiles (height/weight: 3^rd^, 25^th^, 50^th^, 75^th^, 97^th^; BMI: 5^th^, 25^th^, 50^th^, 85^th^, 95^th^) from our age-adjusted sex non-specific curves with those from the CDC 2000 girl and boy curves can be seen in **Supplemental Tables 1-3**.

**Figure 4:**
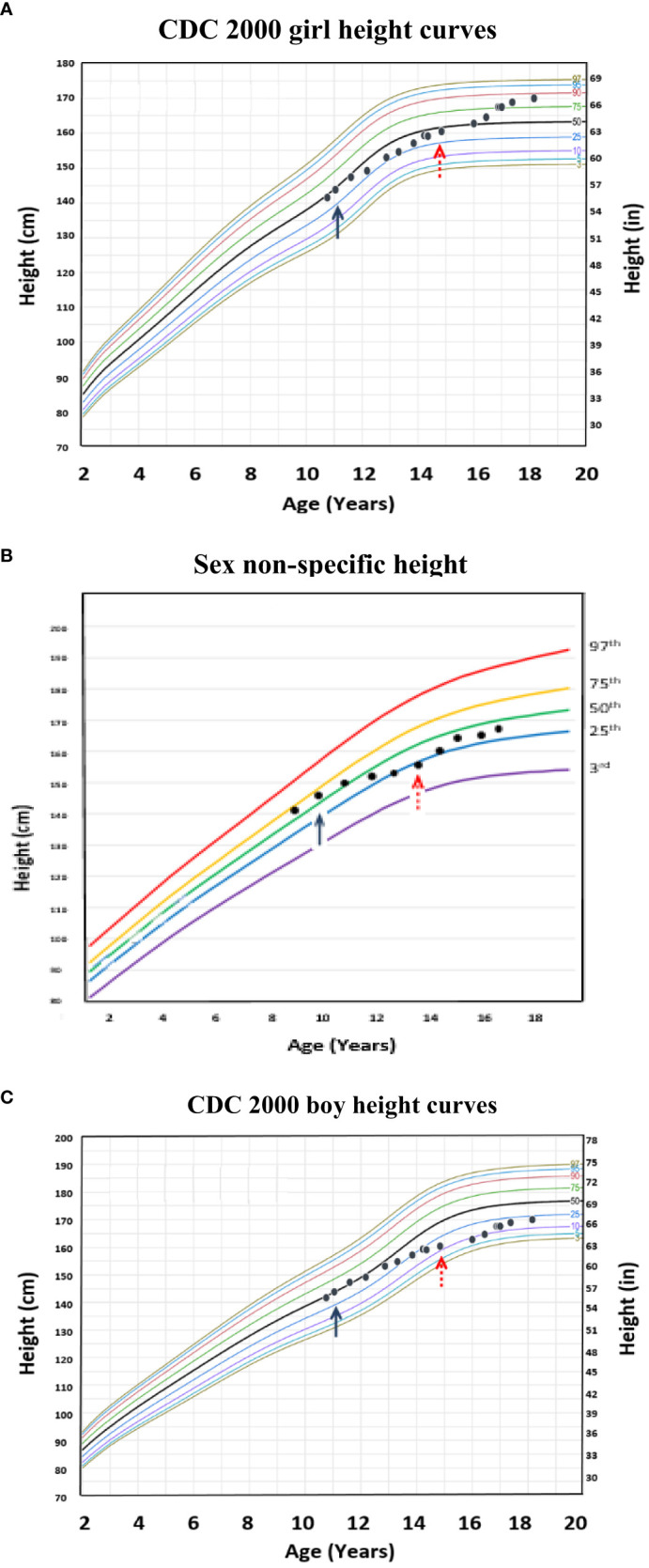
Example of a transgender male (assigned female at birth) individual with height plotted on the age-adjusted sex non-specific height curves. **(B)** shows height plotted on the age-adjusted sex non-specific height curves, while **(A)** and **(C)** show height plotted on the CDC 2000 girl and boy height curves, respectively. This individual received pubertal suppression with gonadotropin releasing hormone agonist therapy (pubertal blocker) beginning around age 12 years (↑) and subsequently began receiving testosterone for cross-sex hormonal therapy beginning around age 15 years (↑).

**Figure 5:**
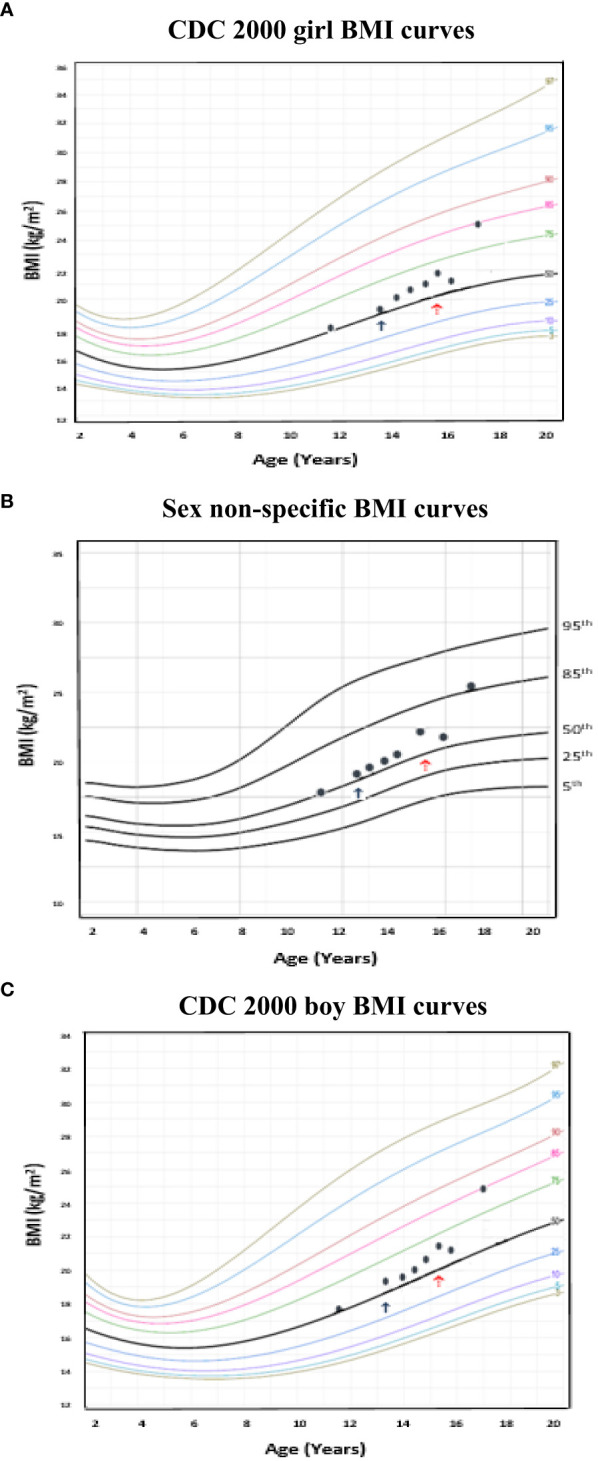
Example of a transgender female (assigned male at birth) individual with body mass index (BMI) plotted on the age-adjusted sex non-specific BMI curves. **(B)** shows BMI plotted on the age-adjusted sex non-specific BMI curves, while **(A)** and **(C)** show BMI plotted on the CDC 2000 girl and boy BMI curves, respectively. This individual received pubertal suppression with gonadotropin releasing hormone agonist therapy (pubertal blocker) beginning around age 13 years (↑) and subsequently began receiving estradiol for cross-sex hormonal therapy beginning around age 15 years (↑). At 17 years old, BMI was in the normal weight category (83rd percentile) on the CDC 2000 girl chart, however, overweight category (86th percentile) on the age-adjusted sex non-specific growth chart.

Although one generally desires to target an appropriate near adult height for one’s chosen gender, this is often an unreasonable expectation, particularly in a transgender male (assigned female at birth) youth. **Figure 4** (transgender male, assigned female at birth, height chart) illustrates how using these sex non-specific charts can be useful in the clinical setting compared to the common practice of comparing male and female charts side-by side for clinical decision-making. **Figure 5** (transgender female, assigned male at birth, BMI chart) illustrates differences in BMI percentiles between the sexes that may lead to misclassification of weight-related disorders in a transgender youth.

Finally, we created age-adjusted sex non-specific z-score calculators for height, weight, and BMI (http://tsaheight2020.shinyapps.io/gender0growthcharts). This website also includes comparative age-adjusted, sex non-specific growth curves, their CDC 2000 girl and boy counterpart curves, and data tables.

## Discussion

Using data from large multi-ethnic cross-sectional populations of US youth, we created age-adjusted, sex non-specific growth charts; reference ranges for height, weight, and BMI; and z-score calculators that age-smooth differences between sexes to assess how a youth is growing “as a child.” While challenging to specifically quantify, and with some differences among height, weight, and BMI, our growth charts are largely superimposable with the CDC 2000 girl and boy charts for about the first 10 years of life, after which divergence becomes more apparent, largely coinciding with general pubertal onset (34, 35). Therefore, clinical utility of these growth charts may have less impact during the pre-pubertal years, however become more important during pubertal onset and thereafter.

While the most commonly used US growth charts have been the WHO (for 0-2 year olds) and CDC 2000 (for 2-19 year olds) charts over the last at least 15 years, clinicians and researchers have realized the necessity of creating additional growth charts to address arisen needs among specific populations. For example, there are now specialized growth charts for Down, Turner, Noonan, and Williams syndromes (36–39). Additionally, pediatric severe obesity (defined as BMI ≥1.2 times the 95^th^ BMI percentile (40)) charts have been generated from CDC 2000 data to better document varying degrees of obesity (41). Of note, creation of these additional charts has largely been made possible through the use of newer statistical techniques and/or larger numbers of youth within these subpopulations available for analysis, and more continue to be developed (31, 32).

The prevalence of youth identifying as transgender has been increasing (42). While difficult to determine, estimates suggest 1.2–4.1% of adolescents report a gender identity different from their genetic sex, with a similar number being variant in gender expression (43). Concurrently, the number of youth presenting for transgender care is growing with a steadily increasing demand for services in multidisciplinary clinics on several continents (6). Methods for tracking growth parameters in this population are lacking, and while it has been proposed that such youth be dually-tracked on girl and boy growth charts, this approach has limitations (i.e., may misclassify diagnoses of weight-related disorders; lead to difficulties predicting near adult height) (7, 10, 44). Therefore, there is a need to track how one is growing as a “transgender child” compared to specifically as a “girl” or “boy.” Our growth charts and z-score calculators can serve as an intermediate reference between the male-specific or female-specific data points until longitudinal growth data are available for the creation of transgender-specific growth charts. Further, as we do not yet know attitudes toward height growth in non-binary young people, sex non-specific charts can serve as a way to monitor overall growth and weight status, and to collect longitudinal data that can be linked to their attitudes/perceptions of growth.

There are a number of clinical applications whereby use of age-adjusted, sex non-specific growth charts may be helpful. For one, differences in BMI percentiles between sexes may misclassify diagnoses of weight-related disorders (i.e., overweight/obesity, underweight) in transgender youth (10). Kidd et al. presents examples illustrating this, including a 16 year old transgender male (assigned female at birth) adolescent on GnRHa and testosterone who would be classified as overweight on the girl but obese on the boy growth chart (10). Similarly, we present (**Figure 5**) a transgender male (assigned female at birth) adolescent who, at 17 years of age had a BMI in the normal weight category (83^rd^ percentile) on the CDC 2000 girls growth chart, however, in the overweight category (86^th^ percentile) on the sex non-specific growth chart. Longitudinal studies linking body composition measures of transgender youth to age-adjusted, sex non-specific BMI percentiles versus BMI percentiles for one’s sex and gender may help to more accurately assess a transgender youth’s weight classification. As the diagnosis of pediatric obesity accompanies medical and psychological sequelae and is associated with increased healthcare utilization, accurate diagnosis is imperative (18, 45–47). Further, if a child or adolescent is not diagnosed with overweight/obesity when they indeed have this, opportunities for earlier intervention and prevention of complications may be missed (18).

We do not believe that use of our age-adjusted, sex non-specific growth charts would impact decisions on when to start pubertal blockade, as early pubertal suppression leads to better psychological and physical outcomes (7). That said, our growth curves may help in guiding medical therapy to potentially augment height. For example, although one generally desires to target an appropriate near adult height for one’s chosen gender, this is often an unreasonable expectation, particularly in a transgender male (assigned female at birth) child during medical intervention with pubertal suppression and cross-hormone sex therapy.

**Figure 4** shows how using these sex non-specific growth charts can be a more practical tool compared to the common practice of comparing the girl and boy charts side-by-side for clinical decision-making in terms of near adult height prediction. Prior to starting puberty blockers at 11 years old, the growth patterns of this transgender male (assigned female at birth) child were similar on the sex non-specific, CDC 2000 boy, and CDC 2000 girl growth charts at around the 50th percentile. However, after three years of pubertal suppression, his growth patterns began to diverge on the CDC 2000 boy and girl charts, a difference that became more pronounced after starting testosterone therapy. On the female chart, growth plots increased from the 25^th^ toward the 50^th^ percentile and, after starting testosterone, further increased to the 85^th^ percentile. On the male chart, growth plots decreased from the 25^th^ to the 10^th^ percentile after starting testosterone, and increased to only the 20^th^ percentile by age 18 years compared to the 85^th^ on the female chart. This level of divergence makes it difficult to assess the impact of testosterone on growth solely by comparing the boy and girl CDC 2000 growth charts. In contrast, on the sex non-specific charts, after three years on pubertal suppression, growth decreased from the 50^th^ to the 25^th^ percentile and, after initiating testosterone, increased back toward the 50^th^ percentile where he was growing prior to pubertal suppression. Therefore, the growth patterns seen on the sex non-specific chart provide an easily interpretable growth trajectory and target for medical interventions that can be used until longitudinal growth charts specific for transgender children are developed.

## Strengths and limitations

Our study has several strengths. Creation of our growth charts and z-score calculators was done using data from large nationally representative multi-ethnic cross-sectional cohorts (19–24). Specifically, we utilized the same datasets and similar statistical techniques used to create CDC 2000 growth charts, allowing for a direct comparison between our charts and those most commonly used in the US (25, 26). Further, our large sample size afforded us adequate statistical power to create these growth charts.

Our study also has limitations. Data used to create our weight and BMI charts were from 1963-1994, largely predating the obesity epidemic. Therefore, these growth charts may be different if more contemporaneous datasets were used. Pediatric severe obesity was rare from 1963-1994, whereas now it is the fastest growing pediatric obesity category (48). In terms of whether the use of more contemporaneous height data would also affect our growth charts (i.e., whether children are now comparatively taller), we suspect this not to be the case based upon current analyses of NHANES data from our group suggesting no secular trends (unpublished). Finally, we note that our z-scores have wider variability compared to those from CDC 2000 resulting from combining girls and boys in our analyses, with differences in pubertal growth spurt timing and long bone growth between sexes.

## Future directions

Although we believe that creation of our age-adjusted, sex non-specific growth charts is an important step towards addressing a critical need in the transgender population, we recognize the imperative need for future research and longitudinal data collection leading to separate growth charts and z-score calculators for transgender male and female youth. This will require significant time and effort. For example, NHANES data for the CDC 2000 growth charts were collected from 1960-1994. Further, it may be helpful to have sex non-specific height velocity charts and z-score calculators given their importance and practicality for tracking changes in height over shorter time durations (49). This is especially important given that height velocity may be altered by GnRHa and/or cross-sex hormonal therapy use (44, 50). Given the inherent limitations of creating height velocity charts using cross-sectional data, development of these charts would be best done using longitudinal cohorts and data registries tracking individuals’ growth over time (51).

## Conclusions

In conclusion, we developed age-adjusted, sex non-specific growth charts that may eventually be used in scenarios in which standard “girls versus boys” growth charts may not be ideal. Presently, our sex non-specific growth charts should be considered a research tool that needs validation before they can be applied to clinical practice. Given the increasing prevalence of youth seeking transgender care and recognized limitations of current approaches, a critical need has arisen in terms of tracking growth parameters in these individuals. Until longitudinal data, including body composition measures, are available in this population, our growth charts may help to assess a transgender youth’s growth trajectory and weight classification, and expectations surrounding this.

## Data availability statement

Publicly available datasets were analyzed in this study. This data can be found here: https://www.cdc.gov/nchs.

## Ethics statement

The studies involving human participants were reviewed and approved by CDC/National Center for Health Statistics institutional review board. Written informed consent to participate in this study was provided by the participants’ legal guardian/next of kin.

## Author contributions

EB contributed to the research concept and design, statistical analyses, and data analysis and interpretation, wrote the first draft of the manuscript, and made critical revisions for intellectual content. BM contributed to the research concept and design, data interpretation, and manuscript write-up, and made critical revisions for intellectual content. OA and MJ contributed to the research concept and design, data interpretation, and manuscript write-up; performed statistical analyses, and made critical revisions for intellectual content. AR contributed to data interpretation, manuscript write-up, and critical revisions for intellectual content. KS developed the research concept and design, contributed to data analysis, interpretation, and manuscript write-up; and made critical revisions for intellectual content. All authors contributed to the article and approved the submitted version.
