# Supplementary material for: Sex non-specific growth charts and potential clinical implications in the care of transgender youth
Source: Front Endocrinol (Lausanne). 2023 Aug 11;14:1227886. doi: 10.3389/fendo.2023.1227886 (PMC10455911; doi:10.3389/fendo.2023.1227886)
Supplement: Supplementary file 1 [file Table_1.docx]

Supplementary Material

Sex Non-Specific Growth Charts and Potential Clinical Implications in the Care of Transgender Youth

Eric M Bomberg*, Bradley S Miller, Oppong Y Addo, Alan D Rogol, Mutaz M Jaber, Kyriakie Sarafoglou

* Correspondence: Eric M Bomberg: [bombe002@umn.edu](mailto:bombe002@umn.edu)

**Supplemental Table 1:** **Comparison of height (cm) between the age-adjusted sex non-specific, CDC 2000 girl, and CDC 2000 boy growth charts for 2 to 20 year olds**

|  | **3^rd^ percentile** | **25^th^ percentile** | **50^th^ percentile** | **75^th^ percentile** | **97^th^ percentile** |
| --- | --- | --- | --- | --- | --- |
| **2 year old** |  |  |  |  |  |
| Sex non-specific | 80.9 | 86.3 | 89.4 | 92.4 | 97.8 |
| CDC 2000 girl | 78.4 | 82.6 | 85.0 | 87.3 | 91.5 |
| CDC 2000 boy | 79.9 | 84.1 | 86.5 | 88.8 | 93.0 |
| **3 year old** |  |  |  |  |  |
| Sex non-specific | 87.6 | 93.2 | 96.3 | 99.3 | 104.9 |
| CDC 2000 girl | 86.9 | 91.6 | 94.2 | 96.9 | 101.8 |
| CDC 2000 boy | 88.4 | 92.7 | 95.3 | 97.9 | 102.9 |
| **4 year old** |  |  |  |  |  |
| Sex non-specific | 94.8 | 100.5 | 103.8 | 107.0 | 112.8 |
| CDC 2000 girl | 93.1 | 98.1 | 101.0 | 104.0 | 109.5 |
| CDC 2000 boy | 94.6 | 99.7 | 102.5 | 105.4 | 110.5 |
| **5 year old** |  |  |  |  |  |
| Sex non-specific | 100.9 | 105.3 | 110.6 | 114.1 | 120.3 |
| CDC 2000 girl | 99.4 | 104.8 | 108.0 | 111.2 | 117.4 |
| CDC 2000 boy | 100.3 | 106.0 | 109.2 | 112.3 | 117.8 |
| **6 year old** |  |  |  |  |  |
| Sex non-specific | 106.6 | 113.4 | 117.1 | 120.9 | 127.6 |
| CDC 2000 girl | 105.8 | 111.6 | 115.0 | 118.6 | 125.3 |
| CDC 2000 boy | 106.1 | 112.3 | 115.7 | 119.1 | 125.1 |
| **7 year old** |  |  |  |  |  |
| Sex non-specific | 112.7 | 119.8 | 123.8 | 127.8 | 134.9 |
| CDC 2000 girl | 111.9 | 118.1 | 121.8 | 125.6 | 132.7 |
| CDC 2000 boy | 112.0 | 118.4 | 122.0 | 125.7 | 132.3 |
| **8 year old** |  |  |  |  |  |
| Sex non-specific | 117.7 | 125.2 | 129.4 | 133.6 | 141.1 |
| CDC 2000 girl | 117.3 | 123.9 | 127.8 | 131.9 | 139.4 |
| CDC 2000 boy | 117.5 | 124.3 | 128.1 | 132.1 | 139.3 |
| **9 year old** |  |  |  |  |  |
| Sex non-specific | 122.6 | 130.6 | 135.0 | 139.5 | 147.4 |
| CDC 2000 girl | 121.9 | 129.0 | 133.1 | 137.4 | 145.4 |
| CDC 2000 boy | 122.4 | 129.6 | 133.7 | 137.9 | 145.7 |
| **10 year old** |  |  |  |  |  |
| Sex non-specific | 127.4 | 136.0 | 140.8 | 145.5 | 154.1 |
| CDC 2000 girl | 126.0 | 133.7 | 138.2 | 142.8 | 151.3 |
| CDC 2000 boy | 126.7 | 134.4 | 138.8 | 143.3 | 151.5 |
| **11 year old** |  |  |  |  |  |
| Sex non-specific | 132.5 | 141.7 | 146.8 | 151.9 | 161.1 |
| CDC 2000 girl | 130.7 | 139.4 | 144.3 | 149.2 | 158.1 |
| CDC 2000 boy | 130.8 | 139.0 | 143.7 | 148.5 | 157.3 |
| **12 year old** |  |  |  |  |  |
| Sex non-specific | 138.3 | 147.6 | 152.9 | 158.1 | 167.5 |
| CDC 2000 girl | 137.4 | 146.5 | 151.5 | 156.4 | 165.2 |
| CDC 2000 boy | 135.7 | 144.3 | 149.3 | 154.4 | 163.7 |
|  | **3^rd^ percentile** | **25^th^ percentile** | **50^th^ percentile** | **75^th^ percentile** | **97^th^ percentile** |
| **13 year old** |  |  |  |  |  |
| Sex non-specific | 144.2 | 153.5 | 158.6 | 163.8 | 173.0 |
| CDC 2000 girl | 144.2 | 152.7 | 157.3 | 162.0 | 170.2 |
| CDC 2000 boy | 141.7 | 151.1 | 156.4 | 161.7 | 171.3 |
| **14 year old** |  |  |  |  |  |
| Sex non-specific | 148.8 | 158.2 | 163.4 | 168.7 | 178.0 |
| CDC 2000 girl | 148.1 | 156.0 | 160.5 | 164.9 | 172.9 |
| CDC 2000 boy | 148.5 | 158.7 | 164.1 | 169.5 | 178.8 |
| **15 year old** |  |  |  |  |  |
| Sex non-specific | 151.3 | 161.2 | 166.7 | 172.2 | 182.1 |
| CDC 2000 girl | 149.7 | 157.5 | 161.9 | 166.3 | 174.2 |
| CDC 2000 boy | 154.6 | 164.8 | 170.1 | 175.3 | 184.1 |
| **16 year old** |  |  |  |  |  |
| Sex non-specific | 151.8 | 162.5 | 168.4 | 174.4 | 185.1 |
| CDC 2000 girl | 150.4 | 158.2 | 162.6 | 166.9 | 174.8 |
| CDC 2000 boy | 158.9 | 168.5 | 173.6 | 178.6 | 187.1 |
| **17 year old** |  |  |  |  |  |
| Sex non-specific | 152.1 | 163.2 | 169.4 | 175.6 | 186.7 |
| CDC 2000 girl | 150.8 | 158.6 | 162.9 | 167.3 | 175.1 |
| CDC 2000 boy | 161.3 | 170.4 | 175.3 | 180.2 | 188.6 |
| **18 year old** |  |  |  |  |  |
| Sex non-specific | 152.3 | 163.4 | 169.6 | 175.8 | 186.9 |
| CDC 2000 girl | 150.9 | 158.8 | 163.1 | 167.5 | 175.3 |
| CDC 2000 boy | 162.5 | 171.3 | 176.2 | 181.0 | 189.5 |
| **19 year old** |  |  |  |  |  |
| Sex non-specific | 152.1 | 163.1 | 169.3 | 175.4 | 186.4 |
| CDC 2000 girl | 151.0 | 158.9 | 163.3 | 167.6 | 175.4 |
| CDC 2000 boy | 163.1 | 171.8 | 176.6 | 181.4 | 189.9 |
| **20 year old** |  |  |  |  |  |
| Sex non-specific | 150.9 | 162.2 | 168.5 | 174.8 | 186.2 |
| CDC 2000 girl | 151.1 | 159.0 | 163.3 | 167.7 | 175.5 |
| CDC 2000 boy | 163.3 | 172.0 | 176.9 | 181.7 | 190.2 |

**Supplemental Table 2: Comparison of weight (kg) between the age-adjusted sex non-specific, CDC 2000 girl, and CDC 2000 boy growth charts for 2 to 20 year olds**

|  | **3^rd^ percentile** | **25^th^ percentile** | **50^th^ percentile** | **75^th^ percentile** | **97^th^ percentile** |
| --- | --- | --- | --- | --- | --- |
| **2 year old** |  |  |  |  |  |
| Sex non-specific | 10.4 | 11.9 | 12.9 | 14.0 | 16.3 |
| CDC 2000 girl | 10.0 | 11.2 | 12.1 | 13.0 | 15.0 |
| CDC 2000 boy | 10.4 | 11.8 | 12.7 | 13.6 | 15.6 |
| **3 year old** |  |  |  |  |  |
| Sex non-specific | 11.8 | 13.5 | 14.7 | 16.0 | 18.9 |
| CDC 2000 girl | 11.4 | 12.9 | 13.9 | 15.2 | 18.0 |
| CDC 2000 boy | 11.8 | 13.4 | 14.4 | 15.6 | 18.1 |
| **4 year old** |  |  |  |  |  |
| Sex non-specific | 13.4 | 15.4 | 16.8 | 18.4 | 22.1 |
| CDC 2000 girl | 12.8 | 14.6 | 15.9 | 17.4 | 21.3 |
| CDC 2000 boy | 13.3 | 15.1 | 16.3 | 17.8 | 21.0 |
| **5 year old** |  |  |  |  |  |
| Sex non-specific | 15.0 | 17.3 | 18.9 | 20.9 | 25.6 |
| CDC 2000 girl | 14.3 | 16.5 | 18.0 | 20.0 | 24.9 |
| CDC 2000 boy | 14.9 | 17.0 | 18.5 | 20.3 | 24.5 |
| **6 year old** |  |  |  |  |  |
| Sex non-specific | 16.7 | 19.3 | 21.2 | 23.6 | 29.5 |
| CDC 2000 girl | 16.0 | 18.5 | 20.3 | 22.7 | 28.9 |
| CDC 2000 boy | 16.5 | 19.0 | 20.8 | 22.9 | 28.3 |
| **7 year old** |  |  |  |  |  |
| Sex non-specific | 18.5 | 21.6 | 23.9 | 26.8 | 34.2 |
| CDC 2000 girl | 17.7 | 20.7 | 22.9 | 25.7 | 33.4 |
| CDC 2000 boy | 18.3 | 21.1 | 23.2 | 25.8 | 32.5 |
| **8 year old** |  |  |  |  |  |
| Sex non-specific | 20.4 | 24.1 | 26.8 | 30.2 | 39.2 |
| CDC 2000 girl | 19.5 | 23.1 | 25.8 | 29.2 | 38.5 |
| CDC 2000 boy | 20.1 | 23.3 | 25.8 | 28.9 | 37.4 |
| **9 year old** |  |  |  |  |  |
| Sex non-specific | 22.5 | 26.8 | 30.1 | 34.2 | 45.5 |
| CDC 2000 girl | 21.6 | 25.9 | 29.1 | 33.3 | 44.6 |
| CDC 2000 boy | 22.1 | 25.8 | 28.7 | 32.4 | 43.1 |
| **10 year old** |  |  |  |  |  |
| Sex non-specific | 24.9 | 30.0 | 33.9 | 38.8 | 52.6 |
| CDC 2000 girl | 24.0 | 29.2 | 33.1 | 38.0 | 51.4 |
| CDC 2000 boy | 24.2 | 28.7 | 32.1 | 36.6 | 49.4 |
| **11 year old** |  |  |  |  |  |
| Sex non-specific | 27.8 | 33.8 | 38.3 | 44.2 | 60.3 |
| CDC 2000 girl | 26.8 | 32.9 | 37.4 | 43.2 | 58.7 |
| CDC 2000 boy | 26.6 | 32.0 | 36.1 | 41.4 | 56.3 |
| **12 year old** |  |  |  |  |  |
| Sex non-specific | 31.1 | 38.0 | 43.2 | 49.9 | 67.9 |
| CDC 2000 girl | 30.0 | 36.8 | 41.8 | 48.3 | 65.9 |
| CDC 2000 boy | 29.5 | 35.9 | 40.7 | 46.8 | 63.3 |
| **13 year old** |  |  |  |  |  |
| Sex non-specific | 34.9 | 42.6 | 48.3 | 55.6 | 74.6 |
| CDC 2000 girl | 33.4 | 40.6 | 46.0 | 53.0 | 72.4 |
| CDC 2000 boy | 33.0 | 40.4 | 45.8 | 52.7 | 70.3 |
| **14 year old** |  |  |  |  |  |
| Sex non-specific | 38.8 | 47.1 | 53.2 | 60.7 | 80.2 |
| CDC 2000 girl | 36.7 | 43.9 | 49.5 | 56.8 | 77.7 |
| CDC 2000 boy | 37.1 | 45.3 | 51.2 | 58.6 | 77.0 |
| **15 year old** |  |  |  |  |  |
| Sex non-specific | 42.1 | 50.8 | 57.1 | 64.9 | 84.6 |
| CDC 2000 girl | 39.6 | 46.7 | 52.1 | 59.4 | 81.7 |
| CDC 2000 boy | 41.5 | 50.2 | 56.5 | 64.2 | 83.2 |
| **16 year old** |  |  |  |  |  |
| Sex non-specific | 44.4 | 53.5 | 60.6 | 68.1 | 88.2 |
| CDC 2000 girl | 41.8 | 48.6 | 54.0 | 61.2 | 84.4 |
| CDC 2000 boy | 45.8 | 54.7 | 61.1 | 69.0 | 89.0 |
|  | **3^rd^ percentile** | **25^th^ percentile** | **50^th^ percentile** | **75^th^ percentile** | **97^th^ percentile** |
| **17 year old** |  |  |  |  |  |
| Sex non-specific | 45.8 | 55.1 | 61.9 | 70.2 | 90.9 |
| CDC 2000 girl | 43.3 | 50.0 | 55.2 | 62.3 | 86.2 |
| CDC 2000 boy | 49.3 | 58.2 | 64.7 | 72.8 | 93.8 |
| **18 year old** |  |  |  |  |  |
| Sex non-specific | 46.4 | 56.0 | 62.9 | 71.5 | 92.8 |
| CDC 2000 girl | 44.3 | 51.0 | 56.2 | 63.4 | 87.4 |
| CDC 2000 boy | 51.7 | 60.7 | 67.3 | 75.6 | 97.3 |
| **19 year old** |  |  |  |  |  |
| Sex non-specific | 46.4 | 56.2 | 63.3 | 72.1 | 94.0 |
| CDC 2000 girl | 44.8 | 51.9 | 57.4 | 64.8 | 88.4 |
| CDC 2000 boy | 53.2 | 62.5 | 69.2 | 77.6 | 99.2 |
| **20 year old** |  |  |  |  |  |
| Sex non-specific | 46.0 | 56.1 | 63.5 | 72.5 | 94.9 |
| CDC 2000 girl | 45.1 | 52.5 | 58.2 | 65.9 | 89.0 |
| CDC 2000 boy | 54.0 | 63.6 | 70.6 | 79.2 | 100.8 |

**Supplemental Table 3: Comparison of body mass index (kg/m^2^) between the age-adjusted sex non-specific, CDC 2000 girl, and CDC 2000 boy growth charts for 2 to 20 year olds**

|  | **5^rd^ percentile** | **25^th^ percentile** | **50^th^ percentile** | **85^th^ percentile** | **95^th^ percentile** |
| --- | --- | --- | --- | --- | --- |
| **2 year old** |  |  |  |  |  |
| Sex non-specific | 14.4 | 15.4 | 16.2 | 17.6 | 18.5 |
| CDC 2000 girl | 14.4 | 15.5 | 16.4 | 18.0 | 19.1 |
| CDC 2000 boy | 14.7 | 15.7 | 16.6 | 18.2 | 19.3 |
| **3 year old** |  |  |  |  |  |
| Sex non-specific | 14.1 | 15.1 | 15.9 | 17.3 | 18.3 |
| CDC 2000 girl | 14.0 | 14.9 | 15.7 | 17.2 | 18.3 |
| CDC 2000 boy | 14.3 | 15.3 | 16.0 | 17.3 | 18.2 |
| **4 year old** |  |  |  |  |  |
| Sex non-specific | 13.9 | 14.8 | 15.6 | 17.1 | 18.2 |
| CDC 2000 girl | 13.7 | 14.6 | 15.3 | 16.8 | 18.0 |
| CDC 2000 boy | 14.0 | 14.9 | 15.6 | 16.9 | 17.8 |
| **5 year old** |  |  |  |  |  |
| Sex non-specific | 13.7 | 14.7 | 15.5 | 17.1 | 18.3 |
| CDC 2000 girl | 13.5 | 14.4 | 15.2 | 16.8 | 18.3 |
| CDC 2000 boy | 13.8 | 14.7 | 15.4 | 16.8 | 17.9 |
| **6 year old** |  |  |  |  |  |
| Sex non-specific | 13.7 | 14.6 | 15.5 | 17.2 | 18.7 |
| CDC 2000 girl | 13.4 | 14.4 | 15.2 | 17.1 | 18.8 |
| CDC 2000 boy | 13.7 | 14.6 | 15.4 | 17.0 | 18.4 |
| **7 year old** |  |  |  |  |  |
| Sex non-specific | 13.7 | 14.7 | 15.6 | 17.6 | 19.2 |
| CDC 2000 girl | 13.4 | 14.5 | 15.5 | 17.6 | 19.7 |
| CDC 2000 boy | 13.7 | 14.7 | 15.5 | 17.4 | 19.2 |
| **8 year old** |  |  |  |  |  |
| Sex non-specific | 13.8 | 14.9 | 15.9 | 18.2 | 20.2 |
| CDC 2000 girl | 13.5 | 14.7 | 15.8 | 18.3 | 20.7 |
| CDC 2000 boy | 13.8 | 14.8 | 15.8 | 18.0 | 20.1 |
| **9 year old** |  |  |  |  |  |
| Sex non-specific | 14.1 | 15.3 | 16.4 | 19.0 | 21.5 |
| CDC 2000 girl | 13.7 | 15.1 | 16.3 | 19.1 | 21.8 |
| CDC 2000 boy | 14.0 | 15.1 | 16.2 | 18.6 | 21.1 |
| **10 year old** |  |  |  |  |  |
| Sex non-specific | 14.4 | 15.7 | 17.0 | 19.9 | 22.9 |
| CDC 2000 girl | 14.0 | 15.5 | 16.9 | 20.0 | 23.0 |
| CDC 2000 boy | 14.2 | 15.5 | 16.7 | 19.4 | 22.2 |
| **11 year old** |  |  |  |  |  |
| Sex non-specific | 14.8 | 16.2 | 17.6 | 20.9 | 24.3 |
| CDC 2000 girl | 14.4 | 16.0 | 17.5 | 20.9 | 24.1 |
| CDC 2000 boy | 14.6 | 15.9 | 17.2 | 20.2 | 23.2 |
| **12 year old** |  |  |  |  |  |
| Sex non-specific | 15.3 | 16.8 | 18.3 | 21.8 | 25.4 |
| CDC 2000 girl | 14.9 | 16.5 | 18.1 | 21.7 | 25.3 |
| CDC 2000 boy | 15.0 | 16.4 | 17.8 | 21.0 | 24.2 |
| **13 year old** |  |  |  |  |  |
| Sex non-specific | 15.9 | 17.5 | 19.0 | 22.6 | 26.2 |
| CDC 2000 girl | 15.3 | 17.1 | 18.7 | 22.6 | 26.3 |
| CDC 2000 boy | 15.5 | 17.0 | 18.4 | 21.9 | 25.2 |
| **14 year old** |  |  |  |  |  |
| Sex non-specific | 16.5 | 18.2 | 19.8 | 23.4 | 26.9 |
| CDC 2000 girl | 15.8 | 17.6 | 19.4 | 23.4 | 27.3 |
| CDC 2000 boy | 16.0 | 17.6 | 19.2 | 22.7 | 26.1 |
| **15 year old** |  |  |  |  |  |
| Sex non-specific | 17.1 | 18.9 | 20.5 | 24.1 | 27.4 |
| CDC 2000 girl | 16.3 | 18.2 | 19.9 | 24.1 | 28.1 |
| CDC 2000 boy | 16.6 | 18.3 | 19.9 | 23.5 | 26.8 |
| **16 year old** |  |  |  |  |  |
| Sex non-specific | 17.6 | 19.4 | 21.0 | 24.7 | 28.0 |
| CDC 2000 girl | 16.8 | 18.7 | 20.5 | 24.7 | 28.9 |
| CDC 2000 boy | 17.1 | 18.9 | 20.6 | 24.2 | 27.6 |
|  | **5^rd^ percentile** | **25^th^ percentile** | **50^th^ percentile** | **85^th^ percentile** | **95^th^ percentile** |
| **17 year old** |  |  |  |  |  |
| Sex non-specific | 17.9 | 19.8 | 21.4 | 25.1 | 28.4 |
| CDC 2000 girl | 17.2 | 19.1 | 20.9 | 25.2 | 29.6 |
| CDC 2000 boy | 17.7 | 19.6 | 21.2 | 24.9 | 28.3 |
| **18 year old** |  |  |  |  |  |
| Sex non-specific | 18.1 | 20.0 | 21.7 | 25.5 | 28.9 |
| CDC 2000 girl | 17.6 | 19.5 | 21.3 | 25.7 | 30.3 |
| CDC 2000 boy | 18.2 | 20.2 | 21.9 | 25.7 | 29.0 |
| **19 year old** |  |  |  |  |  |
| Sex non-specific | 18.2 | 20.2 | 21.9 | 25.8 | 29.2 |
| CDC 2000 girl | 17.8 | 19.7 | 21.6 | 26.1 | 31.0 |
| CDC 2000 boy | 18.7 | 20.7 | 22.5 | 26.4 | 29.7 |
| **20 year old** |  |  |  |  |  |
| Sex non-specific | 18.2 | 20.3 | 22.1 | 26.1 | 29.6 |
| CDC 2000 girl | 17.8 | 19.8 | 21.7 | 26.5 | 31.8 |
| CDC 2000 boy | 19.1 | 21.2 | 23.0 | 27.1 | 30.6 |
